# Clinical and Neuropsychological Phenotyping of Individuals With Somatic Variants in Neurodevelopmental Disorders

**DOI:** 10.1212/NXG.0000000000200254

**Published:** 2025-04-01

**Authors:** Alisa Mo, Christopher A. Walsh

**Affiliations:** 1 Neurology, Boston Children's Hospital, MA;; 2 Genetics and Genomics, Boston Children's Hospital, MA;; 3 Pediatrics, Harvard Medical School, Boston, MA; and; 4 Howard Hughes Medical Institute, Boston Children's Hospital, MA.

## Abstract

**Background and Objectives:**

Somatic variants in brain-related genes can cause neurodevelopmental disorders, but detailed characterizations of their clinical phenotypes, neurobehavioral profiles, and comparisons with individuals with germline variants are limited.

**Methods:**

Using data from the Simons Searchlight natural history cohort, which uses standardized parent-report data collection methods, we identified individuals with neurodevelopmental disorders caused by pathogenic somatic variants and examined their phenotypic data. We further used results from standardized measurements of adaptive functioning, social behavior, and emotional and behavioral problems to compare individuals with somatic variants with those with germline variants.

**Results:**

We identified 15 probands with pathogenic or likely pathogenic somatic variants in the Simons Searchlight cohort. For 8 individuals with detailed phenotype information, symptoms included developmental delay or language delay (n = 8), hypotonia (n = 5), autism spectrum disorder (n = 4), and epilepsy (n = 3). Individuals with mosaic variants showed a range of severity in their scores on standardized measurements of adaptive functioning, social behavior, and emotional and behavioral problems. In particular, some individuals with mosaic variants showed impairments that were similar in severity or more severe compared with individuals with germline variants in the same gene.

**Discussion:**

This study improves our understanding of the clinical phenotypes and neuropsychological profiles of individuals with mosaic pathogenic variants in neurodevelopmental disorders.

## Introduction

Postzygotic variants, also known as somatic or mosaic variants, are acquired after fertilization and lead to the presence of genetically different cell populations in the human body.^1^ Somatic variants that occur early during human development may be common across many tissues, whereas variants that occur later are restricted to specific tissues or even specific cell types within a tissue. The human brain is vulnerable to somatic variants during neurogenesis, and pathogenic somatic variants can cause noncancerous developmental brain lesions including focal cortical dysplasia, hemimegalencephaly, and periventricular nodular heterotopia.^2,3^ These lesional conditions generally lead to epilepsy and varying degrees of global developmental delay, spasticity, autism spectrum disorder (ASD), and other neurologic symptoms. Somatic variants can also contribute to disorders without visible brain lesions, including nonlesional epilepsy, ASD, and intellectual disability.^4,5^ Regarding ASD, studies have estimated that somatic variants contribute to 3%–5% of ASD risk in simplex families.^6-9^

Individuals with somatic variants are generally believed to be less severely affected than individuals with germline variants of the same gene.^10-13^ Some individuals may be asymptomatic or show less severe symptoms^1,14,15^ such as minor dysmorphic features, learning disabilities, or borderline intellectual disability, which are disabilities that are also common in the general population. In some cases, individuals are diagnosed with the somatic variant only after their more severely affected child has been diagnosed with the germline pathogenic variant. Parents with somatic variants in epilepsy-associated genes generally have fewer seizures than their affected children.^16^ Parents with somatic variants without seizures or with few seizures typically had significantly lower variant allele fractions than those with seizures,^17,18^ although there are reports where individuals with somatic variants are also severely affected.^19^ An interesting case is X-linked *PCDH19*-related epilepsy, where only female individuals with germline variants (who show mosaicism due to X-inactivation) and male individuals with somatic variants are symptomatic while hemizygous male individuals are unaffected.^20^ This unique disease transmission pattern is caused by abnormal cell sorting in the brain when both normal and variant neurons are present.^21^ Somatic copy number variations (CNVs) may also result in ASD through different mechanisms compared with germline CNVs.^22^ Small CNVs (e.g., 16p11.2 deletion) need to be in the germline state to cause ASD and have limited phenotypic consequences in the somatic state, presumably because they need to be present in most cells to cause dysfunction. By contrast, large (>4–6 Mb) somatic CNVs significantly increase the risk of ASD and are not found in the germline state, presumably because they lead to spontaneous miscarriage.

Despite the impact of somatic variants on neurologic disease, there are few existing studies that systematically examine the clinical phenotypes and neurobehavioral profiles of individuals with somatic variants in neurodevelopmental disorders. Therefore, the primary goal of this study was to analyze the detailed clinical phenotype and neuropsychological profiles of individuals with somatic variants in neurodevelopmental disorder-related genes compared with their germline counterparts. To achieve this goal, we used the Simons Searchlight research registry to identify individuals with neurodevelopmental disorders who have somatic disease-causing variants and analyzed their clinical phenotyping and performance on standardized measures of adaptive behavior, social communication, and behavioral and emotional problems.

## Methods

### Participants

For both participants with mosaic variants and germline variants, data were obtained from the Simons Searchlight research registry,^23^ previously known as Simons Variation in Individuals Project (Simons VIP) and supported by the Simons Foundation Autism Research Initiative (SFARI). The Simons Searchlight project is an international registry that recruits participants with rare genetic neurodevelopmental disorders and collects variant information as well as detailed cross-sectional and longitudinal medical history. Neuropsychological assessments are also obtained from the family through a structured interview process by a trained genetic counselor. The collection and review of genetic testing reports by Simons Searchlight has been described elsewhere^23,24^ and is briefly summarized here. Families submitted externally performed clinical or research genetic testing reports to Simons Searchlight. Information including the date of testing, type of testing (i.e., exome sequencing, panel sequencing, and chromosomal microarray), testing laboratory, genetic findings, the presence of somatic mosaicism, and evidence applied toward variant classification is extracted from the external laboratory report and entered into the Simons Searchlight database. The classification of whether a variant was somatic or germline was determined by the original testing laboratory and was not independently verified by Simons Searchlight. Genetic testing reports were independently evaluated by the Simons Searchlight genetic counselors to provide an independent variant classification according to American College of Medical Genetics and Genomics variant interpretation guidelines.^24^ Variants of uncertain significance were reviewed annually by Simons Searchlight genetic counselors and could be reclassified. We used probands with pathogenic or likely pathogenic variants in Simons_Searchlight_Dataset_v12.0 and Simons_Searchlight_Single_Gene_Dataset_v8.0 (Table 1). Individuals with variants of uncertain significance or benign variants were excluded. Families with 2 affected siblings and negative parental genetic testing were presumed to be cases of germline mosaicism. All conditions available in the Simons_Searchlight_Dataset_v12.0 and Simons_Searchlight_Single_Gene_Dataset_v8.0 releases were included for analysis.

**Table 1:** Phenotype Information for Individuals With Somatic Variants

Participant	Genetic change	Variant classification	Test	Mosaic fraction	Neurodevelopment	Systemic features
1	*CHD8* [NM_001170629.1:c.3308G>T p.(Gly1103Val)]	Likely pathogenic	ES^a^	Unknown	DD^b^, language delay, ASD, hypotonia, macrocephaly	GERD^c^, constipation, recurrent acute otitis media, immunodeficiency, mast cell activation syndrome
2	*HIVEP2* [NM_006734.3:c.5150dup p.(Leu1718AlafsTer16)]	Pathogenic	ES	23%	DD, language delay, ASD with regression, macrocephaly	Poor feeding, constipation, recurrent acute otitis media, 1 café au lait macule
3	*SCN2A* [NM_021007.2:c.632G>A p.(Gly211Asp)]	Likely pathogenic	Panel	Unknown	DD, hypotonia, epilepsy, microcephaly, CVI^d^	None
4	*SCN2A* [NM_021007.2:c.710T>A p.(Ile237Asn)]	Likely pathogenic	Panel	Unknown	DD, hypotonia, epilepsy, CVI	Poor feeding requiring g-tube, constipation, recurrent acute otitis media, scoliosis
5	*SCN2A* [NM_021007.2:c.4877G>A p.(Arg1626Gln)]	Pathogenic	Panel	Unknown	Unknown	Unknown
6	*SCN2A* [NM_001040143.1:c.4877G>A p.(Arg1626Gln)]	Pathogenic	ES	10%	DD, epilepsy, CVI, nystagmus	GERD, cardiac arrhythmia
7	*STXBP1* [NM_003165.3:c.1217G>A p.(Arg406His)]	Pathogenic	Panel	Unknown	Hypotonia, language delay	Clinodactyly
8	*STXBP1* [NM_003165.3:c.1655G>A p.(Cys552Tyr)]	Likely pathogenic	ES	7%	Unknown	Unknown
9	*SYNGAP1* [NM_006772.2:c.1735C>T p.(Arg579Ter)]	Pathogenic	Panel	22%	DD, language delay, ASD	GERD
10	*SYNGAP1* [NM_006772.2:c.1284T>A p.(Tyr428Ter)]	Pathogenic	Panel	Unknown	DD, language delay, ASD, hypotonia	None
11	*CTNNB1* [NM_001904.3:c. 1041_1044del p.(Val349AlafsTer9)]	Pathogenic	Unknown	Unknown	Unknown	Unknown
12	*IRF2BPL* [NM_024496.3:c. 205_217del; p.(Ser69ArgfsTer79)]	Pathogenic	ES	Unknown	Unknown	Unknown
13	*NEXMIF* [NM_001008537.2:c.2396C>A p.(Ser799Ter)]	Pathogenic	Panel	Unknown	Unknown	Unknown
14	*PPP2R1A* [NM_014225.5:c. 538A>G p.(Met180Val)]	Pathogenic	ES	Unknown	Unknown	Unknown
15	Distal 16p11.2 deletion; arr[hg19]16p11.2(28824490_29043972)x1	Pathogenic	Microarray	Unknown	Unknown	Unknown

a Exome sequencing.

b Developmental delay.

c Gastroesophageal reflux disease.

d Cortical visual impairment.

### Standard Protocol Approvals, Registrations, and Patient Consents

For the Simons Searchlight study, all participants provided written informed consent and the research protocol was approved by the Institutional Review Boards at Geisinger and Columbia University. The authors' access to the deidentified data was approved by the Boston Children's Hospital Institutional Review Board and by Simons Searchlight using SFARI Base. Data were accessed for this article on June 23, 2024.

### Analysis

Clinical and phenotypic information was reviewed for all individuals with somatic likely pathogenic or pathogenic variants in the Searchlight cohort. We used demographic information and medical history information collected by the family through an online parent-reported questionnaire. If more than one time point was collected, then the oldest age was used. Diagnostic conditions such as epilepsy or ASD were documented through parental reports from previous medical professional evaluations.

To assess the neuropsychological phenotype of individuals with somatic variants compared with individuals with germline pathogenic or likely pathogenic variants in the same gene, we used data from structured interviews of the Vineland Adaptive Behavior Scales–Second Edition (VABS-II), the Social Responsiveness Scale–Second Edition (SRS-2), the Social Communication Questionnaire–Lifetime (SCQ), the Child Behavior Checklist for ages 1.5–5 (CBCL/1.5–5), and the Child Behavior Checklist for ages 6–18 (CBCL/6–18). The VABS-II is an assessment of adaptive behavior that is validated over a broad age range (0–60 years).^25^ The VABS-II gives both a total composite score and individual subdomain scores for motor skills, communication skills, daily living skills, and socialization skills. The scores have a mean of 100 and a SD of 15, with higher scores indicating a higher level of adaptive functioning. Although the motor skills domain is an optional section of the VABS-II, only evaluations with available motor skills scores were included for both individuals with somatic and germline variants.

The SRS-2 is a measure of social and other related behaviors and can be used to screen for the risk of ASD.^26^ Total scores and subscale scores in social awareness, social cognition, social communication, social motivation, and mannerisms are reported, with higher normed T-scores indicating greater severity of impairment in social behavior. The SCQ is a screening instrument to evaluate communication skills and social skills in children who are suspected of having ASD.^27^ We used the SCQ–Lifetime form, which focuses on behavior across the participant's entire developmental history. Scores above 15 suggest increased risk of ASD.

The CBCL/1.5–5^28^ and CBCL/6–18^29^ evaluations are caregiver questionnaires used to characterize the scope and intensity of behavioral and emotional problems in preschoolers, children, and adolescents. Broadband scales for internalizing and externalizing symptoms are reported, as well as individual syndrome scales. Raw scores are converted to normed T-scores. For the CBCL/1.5–5 evaluation, there are 7 syndrome scales: emotionally reactive, anxious/depressed, somatic complaints, withdrawn, attention problems, aggressive behavior, and sleep problems. The emotionally reactive, anxious/depressed, somatic complaints, and withdrawn syndrome scales contribute to the broad internalizing problem domain. The attention problems and aggressive behavior syndrome scales contribute to the broad externalizing domain. An additional syndrome scale, sleep problems, is not included in either broad domain, but it is included in the total problems score. For the CBCL/6–18 evaluation, there are 8 syndrome scales: anxious/depressed, withdrawn/depressed, somatic complaints, rule-breaking behavior, aggressive behavior, social problems, thought problems, and attention problems. The internalizing problem domain is composed of the anxious/depressed, withdrawn/depressed, and somatic complaints syndrome scales. The externalizing problem domain is composed of the rule-breaking behavior and aggressive behavior syndrome scales. The total problems score evaluates the overall level of impairment and includes contributions from all 8 syndrome scales.

### Data Availability

The data can be requested directly from the Simons Foundation at sfari.org.

## Results

A total of 2,924 probands were identified to have likely pathogenic or pathogenic variants in neurodevelopmental genes in the Simons Searchlight registry. Fifteen probands (0.5%) had somatic variants. Fourteen of 2,209 participants (0.63%) had somatic single-nucleotide variants while 1 of 715 participants (0.14%) (not significant, chi-square *p* value 0.11) had somatic copy number variants.

While the 15 somatic variants represented a range of genes, 2 participants had the same exact somatic variant (*SCN2A* c.4877G>A; p.Arg1626Gln), which is also a known pathogenic variant when present in the germline. One additional individual had a germline likely pathogenic variant in *ASXL3* and a somatic likely pathogenic variant in *STXBP1*. Because both *ASXL3* and *STXBP1* can lead to neurodevelopmental disability, this individual was excluded from further analysis. Only 4 individuals had reported mosaic fractions, which were 7%**,** 10%, 22%, and 23% (Table 1). These high mosaic fractions are consistent with the relatively modest sensitivity of routine clinical tests to detect somatic variants. Of note, a somatic CNV in 1 individual is a distal 16p11.2 deletion between BP2 and BP3, which is distinct from the classical 16p11.2 deletion between BP4 and BP5 that does not seem to cause ASD symptoms in the mosaic state.^22^

In addition to probands with somatic variants, 20 other probands showed germline variants but had a parent with the same variant in the somatic state, suggesting that these were parents with gonosomal mosaicism, i.e., showing somatic mosaicism for the variant both in germ cells and in blood and hence presumably throughout the body.^1^ There were no developmental data available on most of these parents, but one parent was noted to have a central auditory processing disorder. In addition, there were 23 cases of suspected germline mosaicism, because 2 affected siblings shared the exact same variant, but the variant was not detected in either parent.

We next examined the demographic, genetic, and clinical characteristics of the 15 probands with somatic variants (Table 1). The age of participants ranged from 4 to 73 months, and there were 5 male participants and 9 female participants. A total of 8 participants had additional phenotypic data. Regarding neurologic features, 8 of 8 individuals had a diagnosis of developmental delay and/or language delay. Five of 8 individuals had hypotonia. Half (4/8) of the individuals carried a diagnosis of ASD. Other conditions included epilepsy (3/8 participants), cortical visual impairment (3/8 participants), macrocephaly (2/8 participants), and microcephaly (1/8 participants).

**Figure 1:**
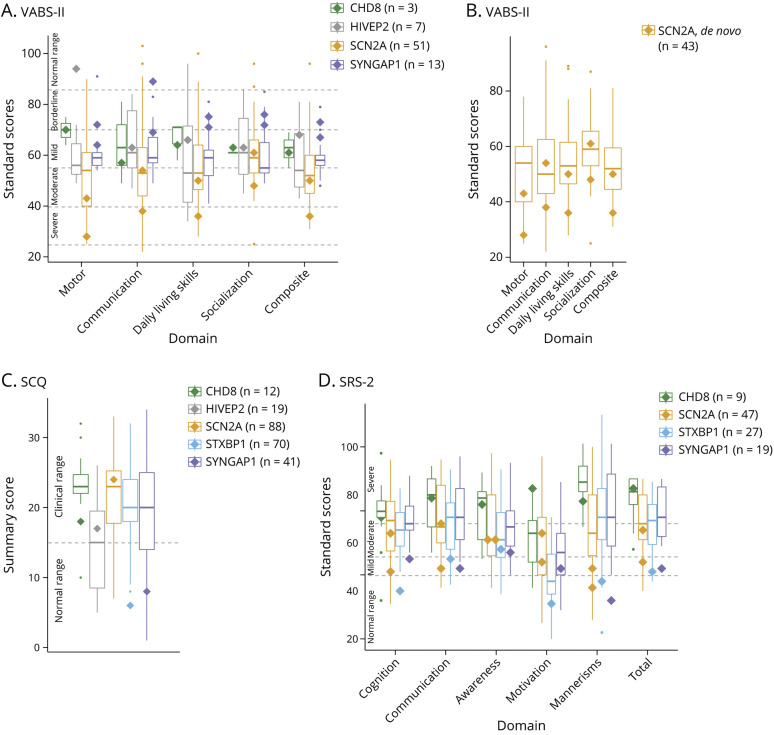
Adaptive Behavior and Social Behavior in Individuals With Neurodevelopmental Disorders Caused by Somatic Variants Compared With Germline Variants The box-and-whisker plot shows the Vineland Adaptive Behavior Scales–Second Edition (VAB-II) standard scores for the composite measurement and individual subdomains (A and B), the Social Communication Questionnaire–Lifetime (SCQ) scores (C), and the Social Responsiveness Scale–Second Edition (SRS-2) T-scores for total and individual subscales (D) for individuals with germline variants for each disease condition. In the box-and-whisker plot, the median (horizontal line within the box), 25th percentile (bottom of the box), 75th percentile (top of the box), minimum values (lower whiskers), maximum values (upper whiskers), and outliers (dots) are shown. The number of participants with germline variants for each gene is listed in parentheses. The scores for the individual(s) with somatic variants for each disease condition are shown by the solid diamonds. Dotted horizontal lines show commonly used levels of impairment. For the VABS-II (A), dotted lines denote 70–84 (−1 to −2 SD) for borderline impairment; 55–69 (−2 to −3 SD) for mild impairment; 40–54 (−3 to −4 SD) for moderate impairment; 25–39 (−4 to −5 SD) for severe impairment; and <24 for profound impairment.^29^ For the SCQ, scores above 15 are more strongly associated with ASD.^30^ For the SRS-2, dotted lines denote 60–65 for mild impairments in social behavior; 66–75 for moderate impairments; and 76 or higher for severe impairments.^26^

To compare the neuropsychological profiles of individuals with somatic variants with those of germline variants in the same gene, we examined standardized measures of adaptive function, social behavior, and emotional/behavioral problems (Table 2). The VABS-II scores showed that individuals with pathogenic somatic variants have a range of adaptive functioning abilities (Figure 1A), including those with scores both above and below those of germline-affected individuals. For example, the 2 participants with somatic *SYNGAP1* variants had better adaptive functioning across all domains compared with most individuals with germline variants in *SYNGAP1*. By contrast, the 2 participants with somatic *SCN2A* variants had similar or worse adaptive functioning across all VABS-II subdomains compared with most individuals with germline variants in *SCN2A*. *SCN2A* variants resulting in increased sodium channel activity (gain-of-function) are associated with early infantile epilepsy with good response to sodium channel blockers, whereas variants resulting in loss-of-function effects are associated with late-onset epilepsy as well as autism and intellectual disability without epilepsy.^31,32^ Because there is a large range of clinical phenotypes in *SCN2A*-related disorders,^19^ we assessed whether the 2 variants (Participant 3: *SCN2A* [NM_021007.2:c.632G>A p.(Gly211Asp)] and Participant 4: *SCN2A* [NM_021007.2:c.710T>A p.(Ile237Asn)]) were known to have severe clinical phenotypes in the germline state. However, we did not find phenotype-genotype information or known functional consequences for these 2 variants. A previous study found that inherited *SCN2A* variants led to milder clinical phenotypes, whereas de novo variants caused more severe phenotypes.^33^ Therefore, we compared the 2 affected individuals with somatic de novo variants with individuals with germline de novo variants in *SCN2A* (Figure 1B). We found that the 2 participants with somatic variants still had similar or worse adaptive functioning than most participants with germline de novo variants.

**Table 2:** Availability of Neuropsychological Data for Individuals With Somatic Variants

Participant	Genetic change	Available testing
1	*CHD8* [NM_001170629.1:c.3308G>T p.(Gly1103Val)]	VABS-II, SCQ, SRS-2, CBCL/6–18
2	*HIVEP2* [NM_006734.3:c.5150dup p.(Leu1718AlafsTer16)]	VABS-II, SCQ, CBCL/1.5–5
3	*SCN2A* [NM_021007.2:c.632G>A p.(Gly211Asp)]	VABS-II, SRS-2, CBCL/1.5–5
4	*SCN2A* [NM_021007.2:c.710T>A p.(Ile237Asn)]	VABS-II, SCQ, SRS-2, CBCL/6–18
7	*STXBP1* [NM_003165.3:c.1217G>A p.(Arg406His)]	SRS-2, SCQ, CBCL/1.5–5
9	*SYNGAP1* [NM_006772.2:c.1735C>T p.(Arg579Ter)]	VABS-II, SCQ, SRS-2, CBCL/6–18
10	*SYNGAP1* [NM_006772.2:c.1284T>A p.(Tyr428Ter)]	VABS-II, CBCL/1.5–5

Participants with somatic variants also showed a range of scores on the SCQ–Lifetime form (Figure 1C), a measure of social and communication skills, and the SRS-2, a measure of social and related behaviors (Figure 1D). Although some participants with somatic variants showed less impairment on these 2 social-related scales compared with their germline counterparts (for example, the individuals with somatic variants in *SYNGAP1* and *STXBP1*), other participants were just as severely affected (for example, the participant with *HIVEP2* somatic variant on the SCQ; the participant with *CHD8* somatic variant on the SRS-2).

**Figure 2:**
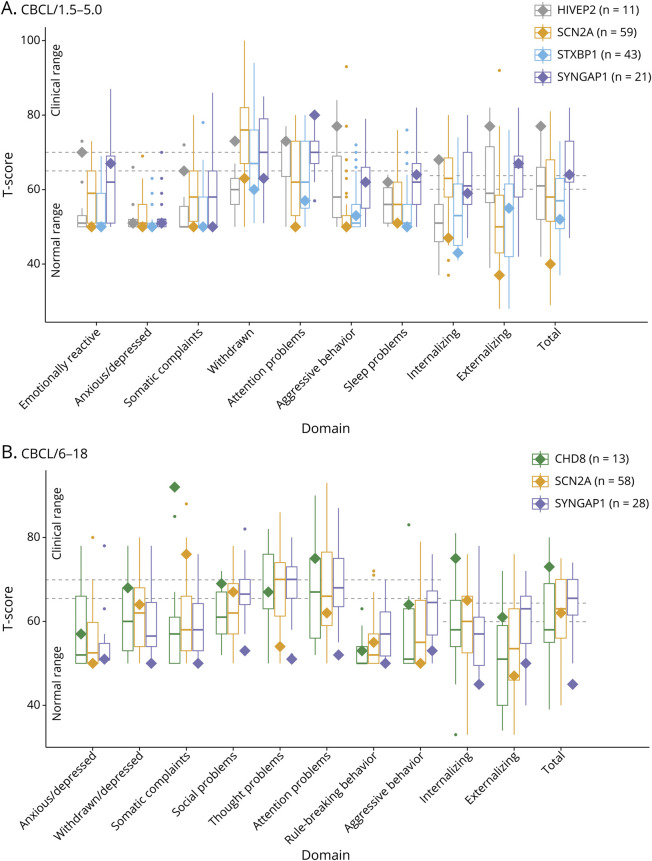
Behavioral and Emotional Problems in Individuals With Neurodevelopmental Disorders Caused by Somatic Variants Compared With Germline Variants The box-and-whisker plot shows the Child Behavioral Checklist for ages 1.5–5 (CBCL/1.5–5) (A) and for ages 6–8 (CBCL/6–18) (B) T-scores for individual syndrome scales, internalizing and externalizing broad domains, and the total problems scale for participants with germline variants for each disease condition. In the box-and-whisker plot, the median (horizontal line within the box), 25th percentile (bottom of the box), 75th percentile (top of the box), minimum values (lower whiskers), maximum values (upper whiskers), and outliers (dots) are shown. The number of participants with germline variants for each gene is listed in parentheses. The scores for the individual(s) with mosaic variants for each disease condition are indicated by the solid diamonds. Dotted horizontal lines show commonly used levels of impairment on the CBCL scales.^28,34^ T-scores 70 or greater are clinically significant in the individual syndrome scales. T-scores 64 or greater are clinically significant on the 2 broadband scales and the total problems scales. Borderline scores (between 65 and 69 on the individual syndrome scales or between 60 and 63 on the broadband scales) are also shown.

Regarding emotional and behavioral symptoms, participants with somatic variants again showed a range of scores in the CBCL/1.5–5 (Figure 2A) and the CBCL/6–18 (Figure 2B) assessments, including scores both above and below the median scores of participants with germline variants. For example, on the CBCL/1.5–5 assessment, individuals with somatic variants in *SCN2A* and *STXBP1* generally showed scores near or below the median scores of individuals with germline variants in their disease. However, despite only having a mosaic fraction of 23%, the participant with mosaic variation in *HIVEP2* showed clinically significant problems in multiple subdomains of the CBCL/1.5–5 (emotionally reactive, withdrawn, attention problems, and aggressive behavior), both the broad internalizing and externalizing problems domains and the total problems domain (Figure 2a). By contrast, the median score for participants with germline variants in *HIVEP2* was only in the clinically significant category for attention problems and was either clinically insignificant or borderline for all other categories.

## Discussion

Overall, participants with disease-causing somatic variants in neurodevelopmental genes showed a spectrum of clinical and neuropsychological phenotypes. As expected, several individuals with somatic variants had less severe phenotypes than reports of patients with germline variants in the same gene. However, our data also demonstrated that some individuals with somatic variants can have impairments in adaptive function, social communication, and behavioral and emotional health that are similar or more severe compared with individuals with germline variants in the same disorder, despite having only a small fraction of cells that carry the pathogenic variant. A key strength of our study is the inclusion of standardized neuropsychological measurements, which allow quantitative and comparative insights into the behavioral profiles of individuals with somatic variants compared with germline variants in the same gene.

Participants with somatic variants with similar or more severe phenotypes than the average participant with germline variants in the same gene could be due to deleterious noncell-autonomous effects in a mosaic individual, such as in the case of *PCDH19.*^21^ There are several limitations in this study. First, it is possible that the somatic variants in this study would also show more severe phenotypes in the germline state, and further phenotype-genotype studies for each variant are needed. Limitations in this report also include the identification of somatic variants. The determination of whether a variant was somatic or germline was conducted by the clinical genetic testing laboratory used by each participant before enrollment into Simons Searchlight, and the type of clinical genetic testing differed across participants. Some participants had exome sequencing, whereas other participants had panel sequencing, which typically sequences each targeted region at higher coverage and, therefore, would be expected to detect mosaic individuals with lower variant allele fractions. Furthermore, customized calling algorithms are important to sensitively identify somatic variants,^6-9^ which are not typically in clinical use. Further analysis using cohorts with deeper genetic sequencing and customized pipelines for identifying and validating somatic variants are needed.

In addition, the mosaic fraction was not available for some of the participants in the study. For participants where the mosaic fraction was available, the mosaic fraction in the samples used for testing (e.g., blood or buccal swab) may not correlate with the mosaic fraction in the brain. This might be especially notable for participants in this study who show macrocephaly, which in many cases reflects effects of the genetic variant in brain progenitor cells. For example, *CHD8* germline variants commonly cause macrocephaly,^35^ and Participant 1 with a somatic *CHD8* variant also showed macrocephaly. This could, therefore, reflect that the *CHD8* variant progenitor cells had a growth advantage, which may in turn result in the variant cells making up a much higher mosaic fraction in the brain than in blood. The study is also limited by the small number of individuals with mosaic variants in the Simons Searchlight database and the availability of clinical and neuropsychological testing information.

In summary, this study describes the clinical phenotyping and neuropsychological profiles of individuals with somatic variants, compared with individuals with germline variants, in neurodevelopmental disorders using standardized medical history questionnaires and measurements of adaptive function, social communication, and behavioral and emotional problems. Our finding that individuals with somatic variants may have neurodevelopmental phenotypes that are just as severe as those with germline variants has implications for family counseling. As sequencing technologies and clinical guidelines for the detection and reporting of somatic variants improve, more individuals with somatic variants will be detected. Further clinical and neuropsychological data from individuals with somatic variants will improve our ability to guide affected families.

## Glossary

ASD: autism spectrum disorder
CBCL/1.5–5: Child Behavior Checklist for ages 1.5–5
CBCL/6–18: Child Behavior Checklist for ages 6–18
CNV: copy number variation
SCQ: Social Communication Questionnaire
SFARI: Simons Foundation Autism Research Initiative
SRS-2: Social Responsiveness Scale–Second Edition
VABS-II: Vineland Adaptive Behavior Scales–Second Edition
